# P-1607. Long COVID and Cardiovascular Diseases Among US Adults: A Survey Study

**DOI:** 10.1093/ofid/ofaf695.1786

**Published:** 2026-01-11

**Authors:** Geoffrey Zhang, John Lin, Dang Nguyen, Madison S McCarthy, Thomas P Giordano

**Affiliations:** Baylor College of Medicine, Houston, TX; University of Pennsylvania, Philadelphia, Pennsylvania; Harvard T.H. Chan School of Public Health, Cambridge, Massachusetts; Yale School of Public Health, New Haven, Connecticut; Baylor College of Medicine, Houston, TX

## Abstract

**Background:**

The burden on people and the healthcare system of persistent COVID symptoms is increasingly being recognized. Although cardiovascular manifestations of long COVID are well-documented, the epidemiologic and disease-specific factors of the bidirectional association remain unclear.

**Figure:**
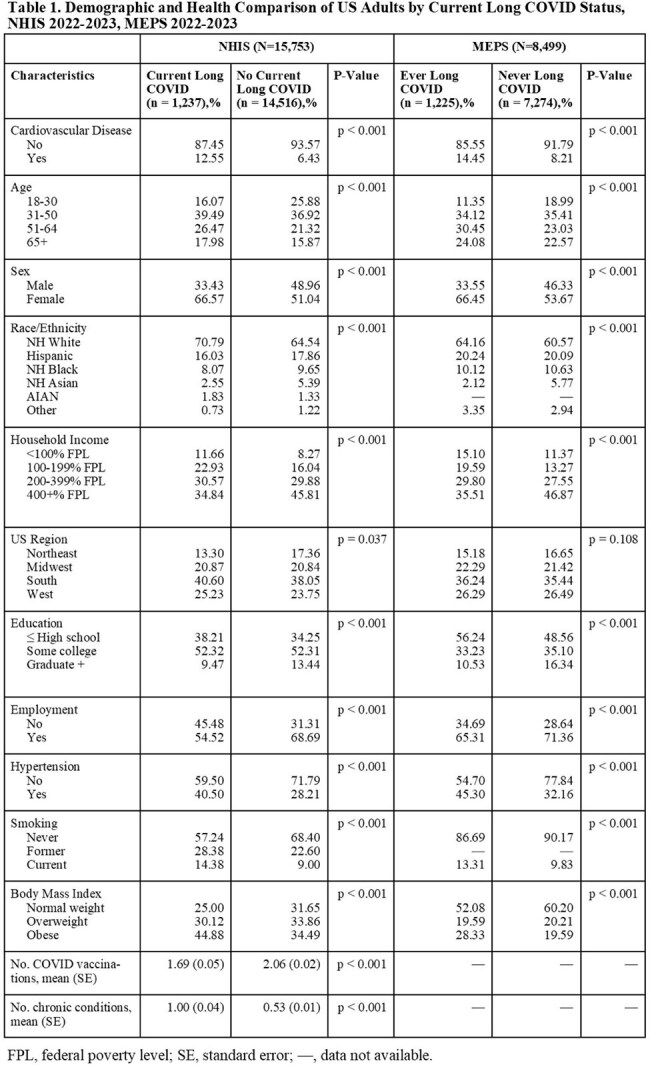

**Figure:**
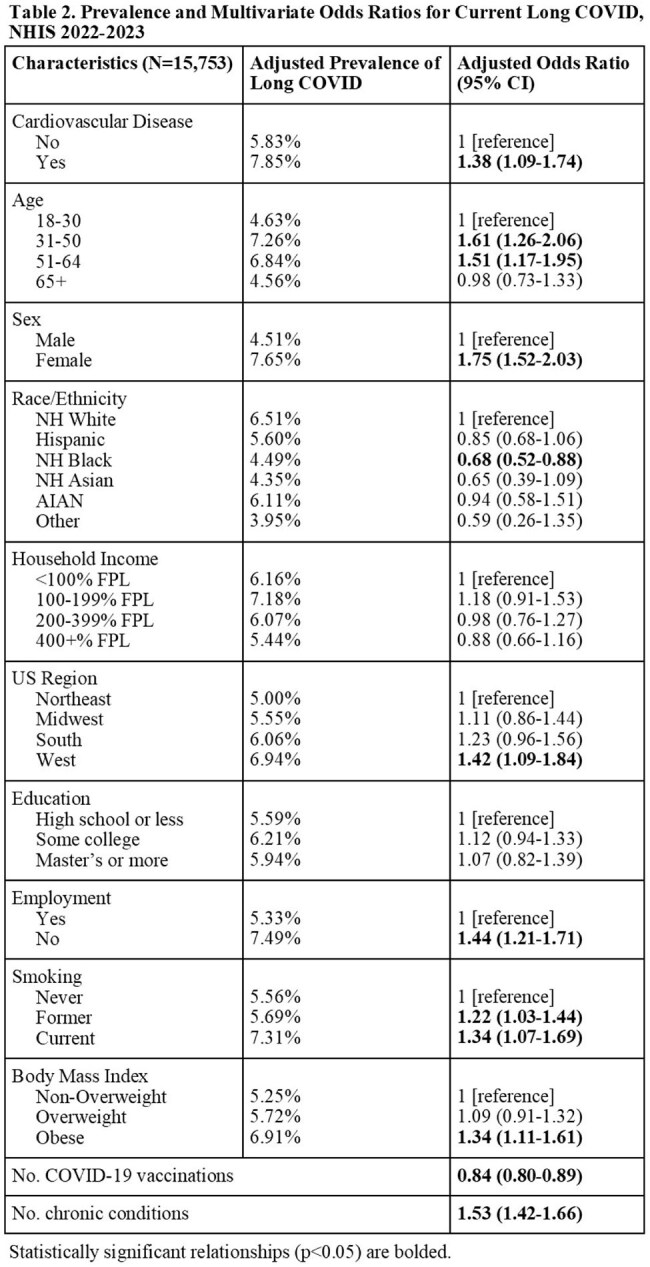

**Methods:**

This study uses nationally representative data from the 2022–2023 U.S. National Health Interview Survey (NHIS) and the 2022-2023 Medical Expenditure Panel Survey (MEPS) to examine the association between long COVID and cardiovascular disease (CVD) in U.S. adults aged 18 years and older. Long COVID was defined as self-reported COVID symptoms persisting beyond three months post-infection. CVD was based on self-reported diagnosis of coronary heart disease, angina, myocardial infarction, or cerebrovascular disease. In NHIS, Rao-Scott χ² and survey-weighted multivariate logistic regression models assessed associations between current and ever long COVID and CVD, adjusting for demographic and socioeconomic factors. In MEPS, temporal relationships were explored by incorporating self-reported age at CVD diagnosis into survey-weighted multivariate logistic regression.

**Figure:**
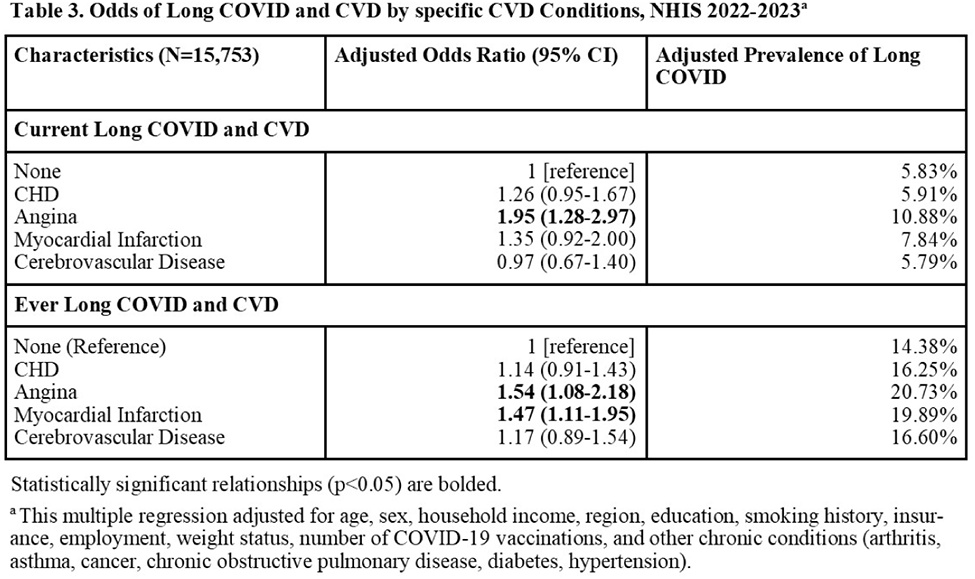

**Figure:**
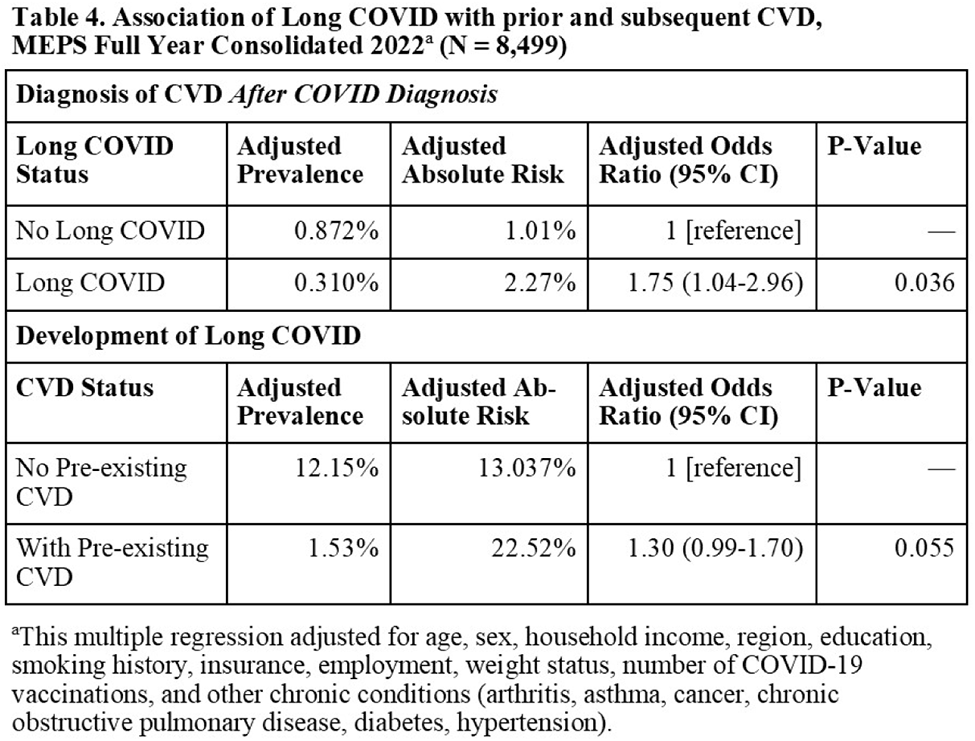

**Results:**

Among 15,753 NHIS respondents (Table 1), 1,237 (7.85%) reported current long COVID. In unadjusted analyses, 12.55% of people with current long COVID reported a CVD diagnosis compared to 6.43% of people without long COVID (p < 0.001). After adjustment in multivariable analysis, current long COVID remained significantly associated with CVD (adjusted OR 1.38; 95% CI: 1.09-1.74; Table 2) and specifically with angina, and myocardial infarction (Table 3). Female sex, obesity, former or current smoking, fewer COVID vaccines and more chronic conditions were also significant correlates. In MEPS (N = 8,499; Table 4), individuals with long COVID had increased odds of a subsequent CVD diagnosis (OR 1.75; 95% CI: 1.04-2.96), and a non-significant trend suggested pre-existing CVD may elevate long COVID risk (OR 1.30; 95% CI: 0.99-1.70).

**Conclusion:**

Our findings demonstrate the impact of long COVID on the development of CVD. Further research to understand the pathophysiology, treatment, and prevention of CVD in people with long COVID is needed to better inform health interventions.

**Disclosures:**

All Authors: No reported disclosures

